# A proposed novel framework for monitoring and evaluation of the cascade of HIV-associated TB care at the health facility level

**DOI:** 10.7448/IAS.20.01.21375

**Published:** 2017-04-20

**Authors:** Colleen F. Hanrahan, Annelies Van Rie

**Affiliations:** ^a^ Department of Epidemiology, Johns Hopkins Bloomberg School of Public Health, Baltimore, MD, USA; ^b^ Epidemiology for Global Health Institute, University of Antwerp, Antwerp, Belgium; ^c^ Department of Epidemiology, University of North Carolina Gillings School of Global Public Health, Chapel Hill, NC, USA

**Keywords:** tuberculosis, HIV, monitoring, cascade of care

## Abstract

**Introduction**: The rapid and accurate diagnosis of HIV-associated tuberculosis (TB), timely initiation of curative or preventative treatment and assurance of favourable treatment outcomes is a complex process. The current system of monitoring and reporting TB diagnosis and treatment does not include several key aspects of the care cascade, and may obscure systematic bottlenecks, inefficiencies or sources of sub-optimal care.

**Methods**: We critically reviewed the current World Health Organizations recommended system of monitoring and reporting, and identified the following key deficiencies that could limit the ability of healthcare workers to identify structural problems in the provision of TB/HIV care.

**Results**: We identified the following key deficiencies in the current monitoring and evaluation system: (1) an emphasis on national-level reporting and programmatic analysis results in a loss of granularity; (2) the absence of a general framework to anchor indicators in relation to one another as well as the overall goals for TB/HIV collaborative activities; (3) de-linking of TB treatment indicators from those for screening and diagnosis; (4) few indicators are tied to suggested times for completion of an activity. We defined three distinct stages comprising the cascade of HIV-associated TB diagnosis and treatment: (1) Screening & Diagnosis, (2) Treatment and (3) Preventive Therapy. We detailed major steps within each stage, described potential sources of variability, and proposed data elements, process indicators, main outcomes, and retention calculations for each stage.

**Conclusions**: This proposed framework of monitoring is novel in its focus on a cohort experience through the entire scope of the care cascade from screening and TB diagnosis through curative or preventive treatment. This approach can be applied to all settings at clinic, district or national level, and used to identify crucial areas for improvement in order to maximize health outcomes for all those affected by the dual epidemics of TB and HIV.

## Introduction

The rapid and accurate diagnosis of HIV-associated tuberculosis (TB), timely initiation of effective treatment (or preventative treatment) and assurance of favourable treatment outcomes is a complex multi-step process, often complicated by inherent resource limitations in the settings where these two epidemics collide. A standardized system for monitoring and reporting of TB diagnosis and treatment was first implemented by the World Health Organization (WHO) in the mid-1990s, as part of the Directly Observed Treatment, Short-course (DOTS) TB control strategy [1,2]. In response to the dual TB/HIV epidemic, in 2004, the WHO published the first version of the Guide to monitoring and evaluation (M&E) for collaborative TB/HIV activities [3]. The document was updated in 2009 and 2015 in order to strengthen the implementation of TB/HIV activities, and improve their quality, and focus on data quality and the utility of the M&E system for the programmatic response.

We critically review the current WHO-recommended tool for monitoring and reporting on HIV-associated TB care and treatment in order to identify gaps. We further explicitly describe the cascade of HIV-associated TB care and propose an enhanced set of indicators for use in routine M&E throughout the diagnostic and treatment pathway of TB/HIV care.

## Discussion

### Critical review of current M & E guidelines

The 2015 guide includes 20 core and 16 optional indicators to monitor and report progress at national level. Broadly, these indicators cover TB screening and diagnosis among HIV-infected individuals, HIV testing among TB cases, TB treatment initiation, preventive treatment (PT) and timely start of antiretroviral treatment (ART). The stated goal of the guidance is to assist TB and HIV programme managers, as well as stakeholders and decision-makers in the monitoring and evaluation of collaborative TB/HIV activities by defining a key set of indicators for national (and global) reporting. The emphasis of evaluation is on aggregation of data from individual health facilities at the district, subnational and national levels. Almost all indicators are expressed as a proportion and are defined at the facility level (e.g. proportion of registered new and relapse TB patients with documented HIV status), though some optional indicators are defined in aggregate (e.g. proportion of health facilities providing TB services that also provide ART services). Strengths of this guide include the detailed description of each indicator, clearly defined numerators and denominators, as well as the rationale, methodology of data collection, data sources, frequency of measurement, and strengths and limitations. Care has also been taken to avoid double-counting in numerators and denominators, and avoid duplication of efforts in data collection by TB and HIV programmes. The indicators are highly valuable for reporting aggregated statistics and making comparisons between districts and countries on basic metrics.

However, several weaknesses exist which reduce the overall potential impact and usefulness of the current M&E system. First, the emphasis on national level reporting and programmatic analysis results in a loss of granularity, which can hinder the identification of the drivers of underperformance or poor outcomes at facility level. For example, a favourable treatment success rate at the national level may obscure high loss to follow-up at individual clinics, where simple interventions such as use of community healthcare workers to trace patients lost to care could be implemented. A second weakness is the absence of a general framework that anchors the indicators in relation to one another as well as the overall goals for TB/HIV collaborative activities. The indicators often “stand alone” from one another, and do not represent the experience of a cohort of individuals along the cascade of diagnosis and care. For example, preventive treatment initiation is often tracked only for individuals with a new HIV diagnosis rather than all patients living with HIV, and a denominator (all patients eligible for IPT) is rarely recorded. The set of “indicators to measure the cascade of intensified TB case finding” represents an effort to capture the flow from HIV testing, through TB screening, diagnosis and treatment initiation, but key elements of this cascade are simplified. For example, TB diagnosis is treated as a binary event when this is in actuality made up of several key steps, including bacteriological testing, follow-up of test-negative individuals, and drug susceptibility testing (DST). Another weakness is the omission of most indicators of TB treatment, as these are covered in the standard TB programme monitoring. This approach de-links the monitoring of screening and diagnosis from treatment monitoring, for which denominators may not align. Finally, with the exception of the indicator “proportion of HIV-positive new and relapse TB patients having profound immunosuppression (CD4 cell count <50) who are started on ART within 2 weeks of TB diagnosis”, none of the indicators are tied to suggested times for completion of an activity. Timing of events is a crucial component of understanding the flow through the TB/HIV care cascade, and its exclusion may hide suboptimal care, such as lengthy time to TB treatment initiation.

In summary, the “Guide to monitoring and evaluation for collaborative TB/HIV activities” offers a valuable set of indicators, particularly at the national and supra-national level. A more holistic approach, with indicators considered as interlinked elements and contextualized within the flow from TB and HIV screening and testing through to treatment (curative or preventive) completion could provide important additional information at facility level.

### The cascade of HIV-associated TB care

**Figure 1.:**
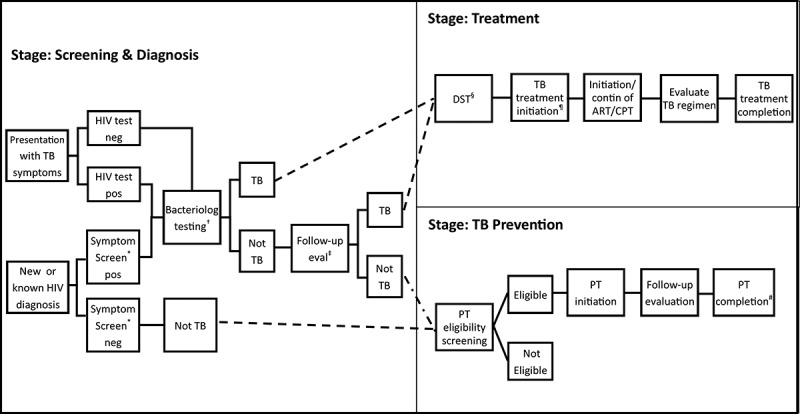
3 stages of the HIV-associated TB care cascade. *TB symptom screening should follow the WHO-recommended approach [4].†Bacteriological testing can include sputum smear microscopy, liquid or solid culture, or a nucleic acid amplification test (such as Xpert MTB/RIF), according to local standard of care and availability.‡Follow-up evaluation can include any combination of additional bacteriological testing, radiology, antibiotic trial or clinical evaluation§DST can be by culture based (liquid or solid), or nucleic acid amplification (e.g. Xpert MTB/RIF, MTBDRplus), and can be conducted on all patients, or targeted to high-risk groups (e.g. retreatment, known MDR contacts)¶TB treatment includes regimens for new or retreatment cases#PT regimen length may vary according to local standard of care.TB, tuberculosis; WHO, World Health Organization; DST, drug susceptibility testing; MDR, multidrug resistant TB; PT, preventive treatment.

We divided HIV-associated TB care into three stages (1) Screening & Diagnosis, (2) Treatment, and (3) Preventive Therapy (Figure 1). The basic components of each stage represent the minimum expectation of integrated TB/HIV care. Different settings or TB control programmes may vary in specific application of these components, either due to in-country availability of specific diagnostic tools, or because of variations in guidelines and practice (e.g. PT eligibility). Below we define each stage, and detail events in the pathway, outcomes, process measures and required data elements.

#### Diagnosis

Individuals feeding into the diagnosis stage arise from two sources: individuals living with HIV or of unknown HIV status presenting with symptoms of TB, and individuals screened for TB symptoms during routine HIV care (Figure 1). Major steps along the diagnostic pathway include HIV testing for those with unknown status, symptom screening for TB in people living with HIV at every HIV-related clinic visit, bacteriologically-based initial testing for all individuals with presumptive TB (i.e. those individuals with signs or symptoms suggestive of TB), and follow-up testing for those negative on the initial diagnostic test. According to WHO guidelines, symptom screening for TB uses the WHO-recommended four-symptom screen [4,5]. Bacteriological testing should at a minimum include sputum smear microscopy, but preferably use a molecular test such as Xpert MTB/RIF, and if possible be followed by sputum culture in those who are smear and/or Xpert MTB/RIF negative [6]. The end point of this diagnosis stage is the individual’s knowledge of TB status as TB or “not TB” [7]. Independent of diagnostic approaches, this outcome should be achieved within a maximum of two months (to accommodate the results from a negative culture), preferably as soon as possible. While this pathway focuses on passive and intensified TB case finding, additional approaches among contacts of known TB cases or other high-risk groups may be applied in some settings. Such strategies would merely expand the population of patients feeding into the diagnosis stage.

The diagnosis stage likely has the most source of variability between settings, as implementation of the four-symptom screen is sub-optimal in most high burden settings [8], and availability of TB diagnostics differs greatly between and within countries. Some settings have a laboratory on-site where bacteriological testing can take place, while others may require sample transport to a centralized laboratory or patient referral to a facility with a laboratory. Follow-up for those testing negative on initial diagnostic testing is likewise not standardized (due in part to a paucity of evidence for best practices) and may vary with diagnostic availability (i.e. smear microscopy only, Xpert MTB/RIF, culture or chest X-ray), level of clinical expertise, and national guidelines or local practices regarding empiric treatment. Indeed, while the goal is to achieve bacteriological confirmation, 42% of all TB diagnoses globally are made on the basis of clinical or radiological findings [9]. We therefore, in accordance with WHO guidelines, stratify TB cases as bacteriologically confirmed or clinical [7]. The timing of the diagnostic stage can also be highly variable, as the entire process could be completed in a single day when using same day microscopy or Xpert MTB/RIF [10–14], or could take weeks or months given the time to negative culture results. Additionally, programmes for TB diagnosis may be physically separated from those where routine HIV care occurs, requiring referral of patients between facilities to complete this stage of the cascade. Finally, we acknowledge the challenge of achieving a “not TB” diagnosis, particularly in settings with limited availability of mycobacterial culture. “Not TB” may include individuals with a definitive alternative diagnosis, as well as those in whom TB has been ruled out.

Estimation of overall retention for this stage requires knowledge of the denominators, that is, total number of people presenting with symptoms of TB and total number of HIV-related clinic visits, as well as the numerators, that is, total number classified as TB, stratified as bacteriologically confirmed and clinical, and total number classified as “not TB”, stratified as definite or presumed (Table 1).

**Table 1.** Stage 1 – Screening and Diagnosis

***Definition***	***Description***
Period from screening for or initial suspicion of pulmonary TB disease until individual receives a diagnosis of TB or “not TB”	This stage encompasses symptom screening, initial TB diagnostic testing, follow-up of those negative on initial test and HIV testing for those of unknown HIV status.
**Measurement of Stage 1 events**	
***Element***	***Proposed measure***
Start point	Individuals presenting with TB symptoms; Individuals presenting for routine HIV care or those with a new HIV diagnosis
Main outcome	Proportion of all presumptive TB with TB diagnosis: bacteriologically confirmed or clinical
Data elements for main outcome	Number of people presenting with TB symptoms, number of people presenting for HIV care, number of newly diagnosed HIV cases, Number of people diagnosed with TB (bacteriologically confirmed or clinical)
Process indicators	Proportion with presumptive TB providing a sample for bacteriological test, proportion sputum results received from laboratory, proportion of referred patients returning with laboratory results, proportion of all patients tested who were positive by initial diagnostic testing, proportion of all patients testing negative who were referred for follow-up testing, proportion of all patients tested with “not TB”, proportion receiving knowledge of positive TB status, time to knowledge of positive TB status
Data elements process indicators	Number of sputum results received from lab, number positive for TB, number referred for and receiving follow-up for negative result, number receiving knowledge of TB status, date of clinic visit, date bacteriological evaluation, date provision of sputum sample, date laboratory results returned to clinic, date of follow-up for bacteriologically negative, number with “not TB” diagnosis, date diagnosis (TB/ “not TB”) communicated to patient
**Retention calculation for Stage 1**	
Number of patients with knowledge of diagnosis (TB or “not TB”)/ number of people presenting with TB symptoms and number of HIV-related clinic visits	

TB, tuberculosis; Xpert, Xpert MTB/RIF.

In addition to the estimation of overall retention, various process indicators can be collected, including the proportion of those with presumptive TB providing a sample for bacteriological testing, proportion of sputum results received from the laboratory, proportion of referred patients returning for results, proportion positive by initial diagnostic, proportion smear/Xpert negative followed-up, and proportion receiving knowledge of TB status (TB or “not TB”).

#### Treatment

The treatment stage encompasses both TB treatment and ART. The starting population consists of individuals who received a TB diagnosis. This stage covers initiation of TB treatment, DST, initiation of ART and cotrimoxazole, and TB treatment monitoring (Figure 1). This stage ends with the documentation of a known treatment outcome, which follows the standard WHO definition of cured, treatment completed, treatment failed, died, loss-to-follow-up and not evaluated [7]. TB treatment outcome applies only to the initiating facility – a patient transferred out to another treatment facility would attain an outcome of “not evaluated” for this stage. Treatment initiation should be achieved within one week of diagnosis, and is a key step in this stage, with implications for both further transmission as well as individual treatment outcomes. Those not initiated within this time frame should be traced as lost-to-follow up. We note that treatment outcome timing will vary by regimen: 6–8 months for drug-sensitive regimen (to incorporate re-treatment or regimen extension) and 9 months or longer for MDR or XDR regimens. Treatment outcomes for MDR-TB as currently recommended by the WHO could be streamlined to facilitate measurement and more accurately reflect true patient outcomes [15,16]. In accordance with WHO guidelines, individuals who successfully complete TB treatment should complete at least six months of PT (Stage 3) [5].

Individual elements of this treatment stage are likely to vary considerably by country and practice setting. DST may be of limited availability, or may be only be applied to select populations based on background prevalence, limited infrastructure, and budgetary restrictions, among other reasons. Differing treatment algorithms, regimens and availability of treatment for drug resistant TB will cause variability in the timing and length of this stage. Despite these differences in practice, most data elements for DST and drug-resistant TB treatment are already included on TB treatment registers, or could be easily incorporated with little added data capture burden. For simplicity, ART initiation and HIV care including cotrimoxazole preventive therapy, a complex cascade itself [17], is treated as a binary step. Monitoring of TB treatment requires assessment of side effects and drug–drug interactions, assessment of clinical and microbiological response to treatment, review of DST results and possible initiation of complementary treatment in case of occurrence of immune reconstitution inflammatory syndrome.

Estimation of overall retention for this stage requires knowledge of the total number of individuals receiving a TB diagnosis at the facility (numerator) and total number of TB patients with known treatment outcomes (inclusive of those not started on treatment) (denominator) (Table 2).

**Table 2.** Stage 2 – Treatment

***Definition***	***Description***
Period from initial patient knowledge of TB diagnosis until treatment outcome	This stage encompasses TB treatment initiation, DST and any necessary changes in treatment or facility, treatment monitoring and completion of treatment
**Measurement of Stage 2 events**	
***Event***	***Proposed measure***
Start point	Patient with TB diagnosis
Main outcome	Known treatment outcome: treatment completion, cure, failure, not evaluated, death, lost to follow-up
Data elements for main outcome	Number of people diagnosed with TB Number of people with outcomes of treatment completion, cure, failure, transfer out, death
Process indicators	Proportion bacteriologically positive patients initiated on treatment, proportion bacteriologically positive patients not started on treatment by 1 week who were traced/contacted, proportion ART-naive TB patients initiated on ART, proportion TB patients initiated on CPT, proportion of all TB cases assessed by DST, proportion of all those assessed by DST with DST results received from lab, proportion all TB cases started on treatment receiving TB treatment monitoring, proportion bacteriologically positive patients who remain positive at conclusion of intensive phase,, time to treatment start, proportion bacteriologically positive patients who remain positive at conclusion of treatment
Data elements for process indicators	Number starting treatment, number started on ART and/or CPT, number assessed by DST, number receiving DST results, number receiving treatment adjustment from DST, number receiving treatment monitoring, number lost to follow-up, date of treatment initiation, date of sample collection for DST, date DST result available, date of sample for treatment monitoring, number bacteriologically positive at end of intensive phase, number bacteriologically positive at end of treatment, date of outcome ascertainment
**Retention calculation for Stage 2**	
Number of people with TB diagnosis/ (number of people with treatment outcome +number of people with TB diagnosis not started on treatment)	

TB, tuberculosis; ART, antiretroviral treatment; CPT, cotrimoxazole preventive treatment; DST, drug susceptibility testing.

#### Preventive therapy

The preventive therapy stage follows after a diagnosis of “not TB”, or after successful completion of TB treatment PT eligibility is assessed, and duration of PT is determined. This stage ends with documented completion of the PT regimen. Initiation of PT should occur within one month of being assessed as eligible, though the timing of completion will depend on local PT guidelines. Current WHO PT guidelines specify that people living with HIV should be screened for TB at every clinical visit, and that those screening negative should be offered IPT. Ideally, PT should be initiated the same day as a negative screen, but we allow that high patient volumes, clinician shortages and other demands may limit what is accomplished at a single consultation. We therefore chose one month as it represents typical visit spacing, particularly early in HIV care and treatment, and believe that one month represents the maximum amount of time for which a negative symptom screen could be valid, as any longer time period would require a new screen.

PT guidelines may vary between settings in terms of eligibility, regimen, and duration. Completion of PT can be based on prescription pick up, but may also incorporate patient self-report of adherence or pharmacy records. As with Stage 2 (Treatment), ART and CPT initiation or continuation are included as a single simplified step.

Estimation of overall retention for this stage requires knowing the total number of individuals who are eligible for PT (denominator) and the total number completing their PT regimen (nominator) (Table 3).

**Table 3.** Stage 3 – Preventive Therapy

**Definition and description of Stage 3**	
***Definition***	***Description***
Period from patient knowledge of “not TB” to completion of PT regimen	This stage encompasses screening for PT eligibility, initiation and completion of PT
**Measurement of Stage 3 events**	
***Event***	***Proposed measure***
Start point	Patient with “not TB”
Main outcome	PT completion
Data elements for main outcome	Number of people diagnosed with “not TB”, number of people eligible completing PT
Process indicators	Proportion all TB screen-negative patients screened for PT eligibility, proportion eligible for PT, proportion of all eligible patients initiating PT, time to start of PT, proportion of all potentially PT eligible, ART-naive initiated on ART, proportion of all potentially PT eligible initiated on CPT,
Data elements for process indicators	Number screened for PT eligibility, number eligible, number initiating PT, date of PT eligibility screening, date start PT, date end PT, number starting treatment, number started on ART and/or CPT
**Retention calculation for Stage 3**	
Number completing PT course/Number eligible for PT	

TB, tuberculosis; PT, preventive therapy.

### Data collection considerations

A comprehensive system of monitoring and evaluation such as we have proposed here not only facilitates an understanding of the successes and challenges programmatic implementation and assists in identifying areas which require improvement, but also promotes accountability within the health care system. Ideally, this system would be implemented routinely in settings where TB and HIV care occur, with at least annual standardized analysis of both main outcomes and process indicators. Indeed many of the data elements proposed herein are already routinely captured. Through simple modifications of standardized logs and registers, such as the sputum collection log, additional elements could be added in to systems which are already in place. We acknowledge, however, that collection of good-quality data can be time consuming and burdensome when systems are not seamless, or when duplication occurs through overlapping programs. Further, scarce resources may limit the ability to provide consistent and quality data monitoring in some settings. In these cases, we propose that this system is implemented periodically with the assistance of a dedicated data capturing team, and perhaps targeted only to problematic districts or facilities. Where possible, once problems have been identified, a follow-up round of monitoring can occur in order to measure progress.

## Conclusions

The WHO recently released ambitious 2025 targets for TB control, including halving TB incidence and reducing TB deaths by 75% [18]. Although new tools are needed for rapid, accurate diagnosis, treatment shortening and active case finding, we cannot ignore that timely diagnosis, rapid initiation and completion of treatment, and implementation of preventive treatment using current tools can offer significant gains and hasten progress towards these goals [19,20]. Current methods for evaluating TB programmes are not sufficient to identify gaps or areas for improvement, especially at facility level. Indeed, a systematic review of TB and HIV service integration at the clinic level found that there is a paucity of data available through which one can understand the performance and best practices of these programmes [21]. Furthermore, monitoring TB/HIV activities and TB treatment as well as the different stages of TB/HIV care are often undertaken separately, and do not consider the entire cascade from initial screening for TB through to completion of curative or preventive treatment nor the timing of any of these activities, making it impossible to understand which steps of the complex pathway are bottlenecks towards favourable health outcomes.

We aimed to develop a more holistic, integrated system for monitoring of HIV-associated TB care that draws a focus to the integration of diagnosis, treatment and prevention as related, rather than separate programmatic goals. We hope this will stimulate discussion and debate on the precise data elements and outputs that can enhance monitoring of the process in order to improve patient outcomes, but do not place an undue burden on care providers and public health workers. We envision this framework to be implemented and interpretable at the individual facility level, where setting-specific problems need to be identified and acted upon. Data could then also be aggregated at the district and national level to facilitate comparison between programmes and identify broad areas for investment and improvement.
